# Changes in the epidemiology of hepatitis A in three socio-economic regions of China, 1990–2017

**DOI:** 10.1186/s40249-019-0591-z

**Published:** 2019-10-03

**Authors:** Xiao-Jin Sun, Guo-Min Zhang, Rong-Jun Zhou, Hui Zheng, Ning Miao, Zun-Dong Yin, Fu-Zhen Wang

**Affiliations:** 1 National Immunization Programme, Chinese Center for Disease Control and Prevention, No. 27 Nanwei Road, Beijing, 100050 China; 2 Guangxi Center for Disease Control and Prevention, Nanning, China

**Keywords:** Hepatitis a, HepA epidemiology, HepA incidence, Coverage, Socioeconomic, Regions

## Abstract

**Background:**

Hepatitis A (HepA) vaccination and economic transitions can change the epidemiology of HepA. China’s Gross Domestic Product (GDP) per capita was known to be inversely associated with the incidence of HepA, but a deeper understanding of the epidemiology of HepA in different socio-economic regions is lacking. We compare the changing epidemiology of HepA in three socioeconomic-geographic regions of China.

**Methods:**

We obtained data on all HepA cases reported through the National Notifiable Disease Reporting System and assessed trends and changes in age-specific incidence rates by age quartile and season. We categorized the country into three regions, the sequential years into five era, compared the incidence, quartile age, seasonal intensity and coverage of HepA of the three regions. Linear regression was performed to analyse trends in incidence of HepA and to analyse the association between coverage and incidence.

**Results:**

The annual mean incidences of HepA in the eastern, central, and western regions decreased from 63.52/100 000, 50.57/100 000 and 46.39/100 000 in 1990–1992 to 1.18/100 000, 1.05/100 000 and 3.14/100 000 in 2012–2017, respectively. Decreases in incidence were seen in all age groups in the three regions; the incidence was highest (9.3/100 000) in the youngest age group (0–4 years) of the western region, while in the central region, the age group with the highest incidence changed from 0 to 9 years to adults ≥60 years old. In 2017, the median age of HepA cases was 43 years (Q_1_–Q_3_: 33–55), 47 years (Q_1_–Q_3_: 32–60) and 33 years (Q_1_–Q_3_: 9–52) in the eastern, central, and western provinces, respectively. Seasonal peaks became smaller or were nearly elimination nationwide, but seasonality persisted in some provinces. After the Expanded Program on Immunization (EPI) included HepA vaccine into the routine schedule in 2007, HepA coverage increased to > 80% in the three regions and was negatively association with the HepA incidence.

**Conclusion:**

The incidence of HepA decreased markedly between 1990 and 2017. A socioeconomic inequity in coverage of HepA vaccine was almost eliminated after HepA vaccine was introduced into China’s EPI system, but inequity in incidence still existed in lower socio-economic developed region.

**Electronic supplementary material:**

The online version of this article (10.1186/s40249-019-0591-z) contains supplementary material, which is available to authorized users.

## Multilingual abstracts

Please see Additional file 1 for translations of the abstract into the five official working languages of the United Nations.

## Introduction

Hepatitis A (HepA) is the frequent causes of food-borne infection in China, causing sporadic cases, outbreaks or epidemics [1]. An epidemic in Shanghai in 1988, associated with eating raw clams, resulted in more than 300 000 cases of HepA and 8000 hospitalizations [2]. Hepatitis A virus (HAV) epidemiology is closely associated with socio-economic development, and poses significant risk for countries with transitioning economies [3, 4]. In the 1980s, the National People’s Congress categorized provinces of the mainland of China into three regions - eastern, central, and western - signifying high (eastern) to low (western) socio-economic development (see Additional file 2). With economic expansion, GDP per capita increased rapidly in all three regions, but with significant differences in rate of increase.

The National Notifiable Disease Reporting System (NNDRS) is a hospital-based, national, passive surveillance system that covers all county and township hospitals in mainland China. NNDRS was established in 1990 and became web-based in 2004. HepA has been continuously reportable since the start of NNDRS. Two types of HepA vaccine are used in China. A live attenuated, 1-dose HepA vaccine has been available since 1992, with more than 15 million doses released annually, produced by four manufacturers. An inactivated, 2-dose HepA vaccine has been available since 2002 and is produced by three manufacturers [5]. Live attenuated HepA is more widely used, as 27 of the 31 provinces use this vaccine while the other 4 provinces use the inactivated HepA vaccine. Coverage of targeted children increased over the years of vaccine availability [6]. In addition to vaccination, improvements in safe water supplies, food safety, sanitation, and hand washing have been changing the epidemiology of HepA in China [7, 8] and other countries [4, 9].

Previous studies have shown that per-capita GDP is inversely associated with HepA incidence [10], but no evaluations of changes in the epidemiology of HepA in different national regions have been conducted. We report a longitudinal study that compares the epidemiology of HepA in the three socioeconomic regions on China’s mainland.

## Methods

### Data sources

All cases of HepA reported through NNDRS from January 1, 1990 to December 31, 2017 were included in this study - both laboratory-confirmed and clinically-diagnosed cases. The ages of individual cases reported between 1990 and 2003 were not available.

Vaccination status of the population under 30 years old was assessed in a national HBV serological survey that was conducted in 2014. Individuals’ vaccination statuses were determined from vaccination cards of sero-survey respondents or by respondent recall [11]. National, regional, and subgroup demographic data were obtained from China Statistical Yearbooks compiled by the National Bureau of Statistics.

We used the following case definitions. A clinically-diagnosed case had abrupt onset with fever, headache, nausea, extreme fatigue, anorexia, vomiting, diarrhea, dark urine, clay-colored stools, and jaundice, and a two-fold or above increase of alanine aminotransferase (ALT). A laboratory-confirmed case was a clinically-diagnosed case with a positive immunoglobulin M test for antibody to HepA virus (anti-HAV) in the absence of recent vaccination.

### Data analysis

In mainland of China, the eastern region included nine highly developed coastal provinces/ municipalities; the central region included 10 provinces; the western region included 12 less-developed provinces/autonomous regions/municipalities. The study years were categorized as the pre-HepA vaccine era (1990–1992); the years when HepA vaccine was available but NNDRS was not upgraded to include age (1993–2003); the years after NNDRS was upgraded but before HepA vaccine was included as a free vaccine in the EPI system (2004–2007); the first 4 years of HepA being included in EPI (2008–2011); and the six most recent years (2012–2017).

We compared the incidence of HepA in the three regions, using incidence rate per 100 000 total population. Linear regression was performed to analyse trends in incidence of HepA and to analyse the association between coverage and incidence [12]. If 95% confidence intervals (*CI*) of linear regression coefficients overlapped, we consider them to be not significantly different. Age was categorized into 0–4, 5–9, 10–19, 20–29, 30–39, 40–49, 50–59 and ≥ 60 years. We calculated the quartiles of ages of cases from 2004 to 2017 in the three regions (age was not available in NNDRS until 2004). Seasonal intensity of HepA was determined applying the improved Muster method, with concentrations from 0 to 100, representing low to high seasonality [13]. We compared coverage of HepA vaccine of the three regions. Data were analysed using SAS software (version9.4, SAS Institute, Inc., Cary, NC, USA), and Microsoft Excel (version 2016, Microsoft, Inc., Redmond, USA).

### Ethical aspects

Ethical approval was not required as analysis of NNDRS data is routine public health work. Individual-identifying information was not available and therefore not used.

## Results

### HepA from 1990 to 2017

**Fig. 1:**
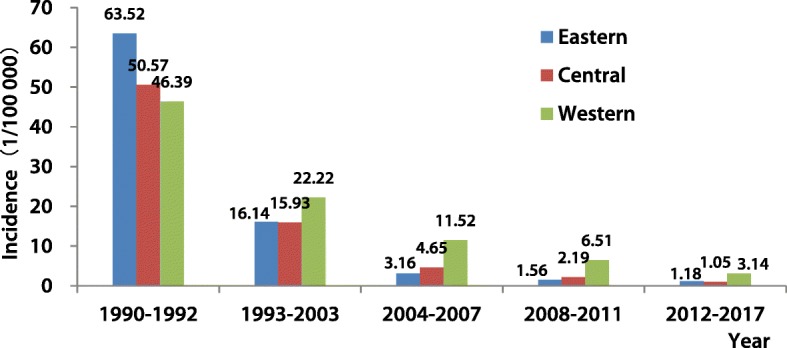
Incidence of hepatitis A in the three regions of China, 1990–2017

From 1990 to 2017, 4 844 438 cases of HepA were reported to NNDRS. During the pre-HepA vaccine phase in China (1990–1992), the mean annual incidence was 53.45/100000, with a higher incidence in the eastern region (63.52/100000). After 1992, incidence decreased in all three regions, with the highest incidence shifting from the eastern region to the western region. Compared with 1990–1992, the incidence in 2012–2017 declined 62.34, 49.52, and 43.25 fold in the eastern, central and western regions, respectively (Fig. 1). The linear regression analyses coefficients between annual incidence and year were − 1.98 (95% *CI*: − 2.58 to − 1.38), − 1.64 (95% *CI*: − 2.09 to − 1.26) and − 1.56 (95% *CI*: − 1.84 to − 1.29) for the eastern, central, and western regions. The 95% *CI*s overlapped, indicating no significant difference.

### Age-specific incidence, 2004 to 2017

During 2004–2007, the HepA incidence among younger age groups in the eastern region (< 2/100000 among 0–19 years) was lower than among older age groups. However, the incidence among young age groups was extremely high in the central and western regions, much higher than among the older age groups. During 2008–2011, incidence rates declined in all age groups in the three regions, decreasing to < 1/100000 in the eastern region among 0–19 years. The incidence remained higher in the central and western regions.

**Fig. 2:**
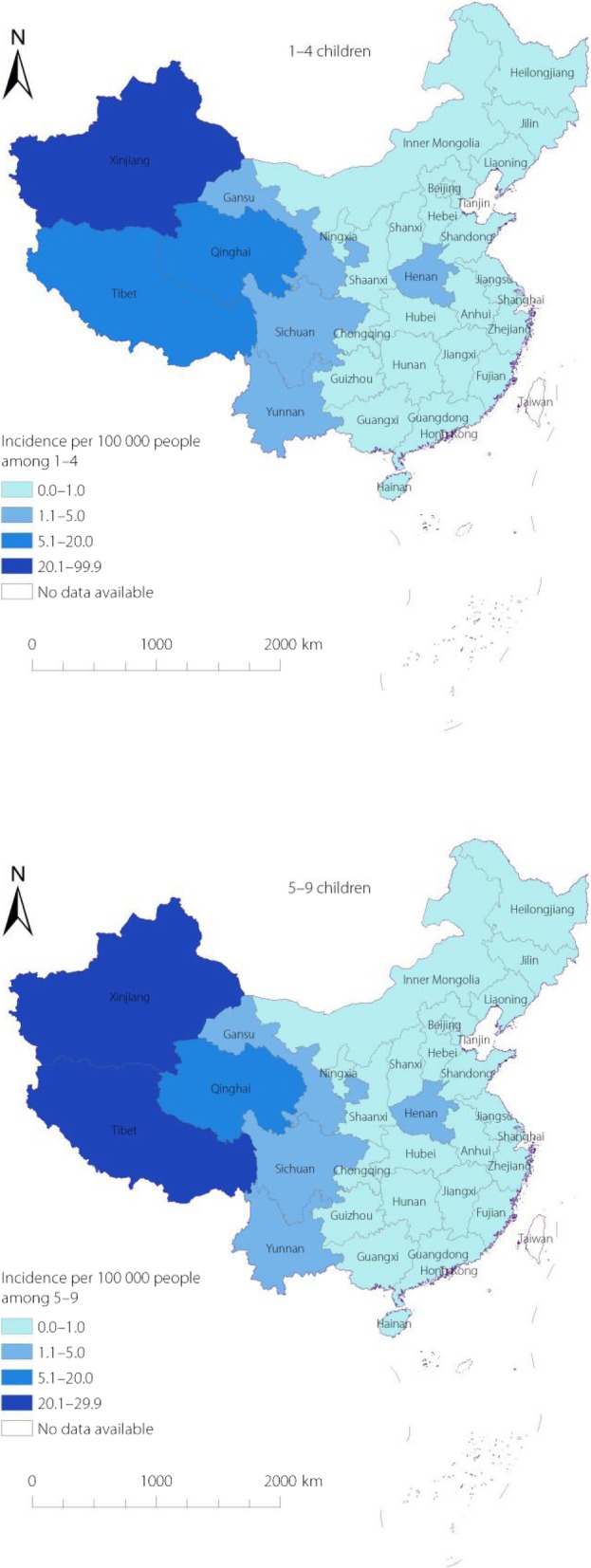
Incidence of HepA among 0–4 and 5–9 children in 2012–2017, China

During 2012–2017, incidence rates were continuously decreasing in all age groups. The incidence among 0–29 years was < 1/100000 in the eastern and central regions, but was higher in the western region. In the western region, incidence rates in all age groups were higher than in the eastern and central regions, and the incidence among 0–4-year-old children (9.26/100000) was highest (Table 1). The incidence among under 10 years old was still high in some western provinces, such as Xinjiang, Qinghai, and Tibet (Fig. 2).

**Table 1:** Age-specific incidence of hepatitis A in the three regions of China, 2004–2017

Age groups	2004–2007			2008–2011			2012–2017		
(Year)	Eastern	Central	Western	Eastern	Central	Western	Eastern	Central	Western
0–4	0.8	6.1	16.5	0.5	2.8	10.8	0.3	0.6	9.3
5–9	1.6	12.0	28.3	0.8	4.3	15.5	0.2	0.6	4.3
10–19	1.4	4.1	13.2	0.6	1.7	7.9	0.3	0.5	2.3
20–29	3.9	3.4	10.3	1.7	1.5	5.8	0.8	0.7	2.3
30–39	4.1	3.8	9.7	2.3	1.9	5.1	1.7	1.0	2.5
40–49	3.8	4.3	8.0	2.0	2.2	4.7	1.5	1.1	2.6
50–59	3.5	4.7	8.2	1.4	2.3	4.3	1.5	1.5	3.1
≥ 60	3.6	4.6	7.8	1.7	2.5	4.7	1.5	2.0	3.7

Note: Incidence was 1/100000

### Quartile ages of HepA cases, 2004 to 2017

**Fig. 3:**
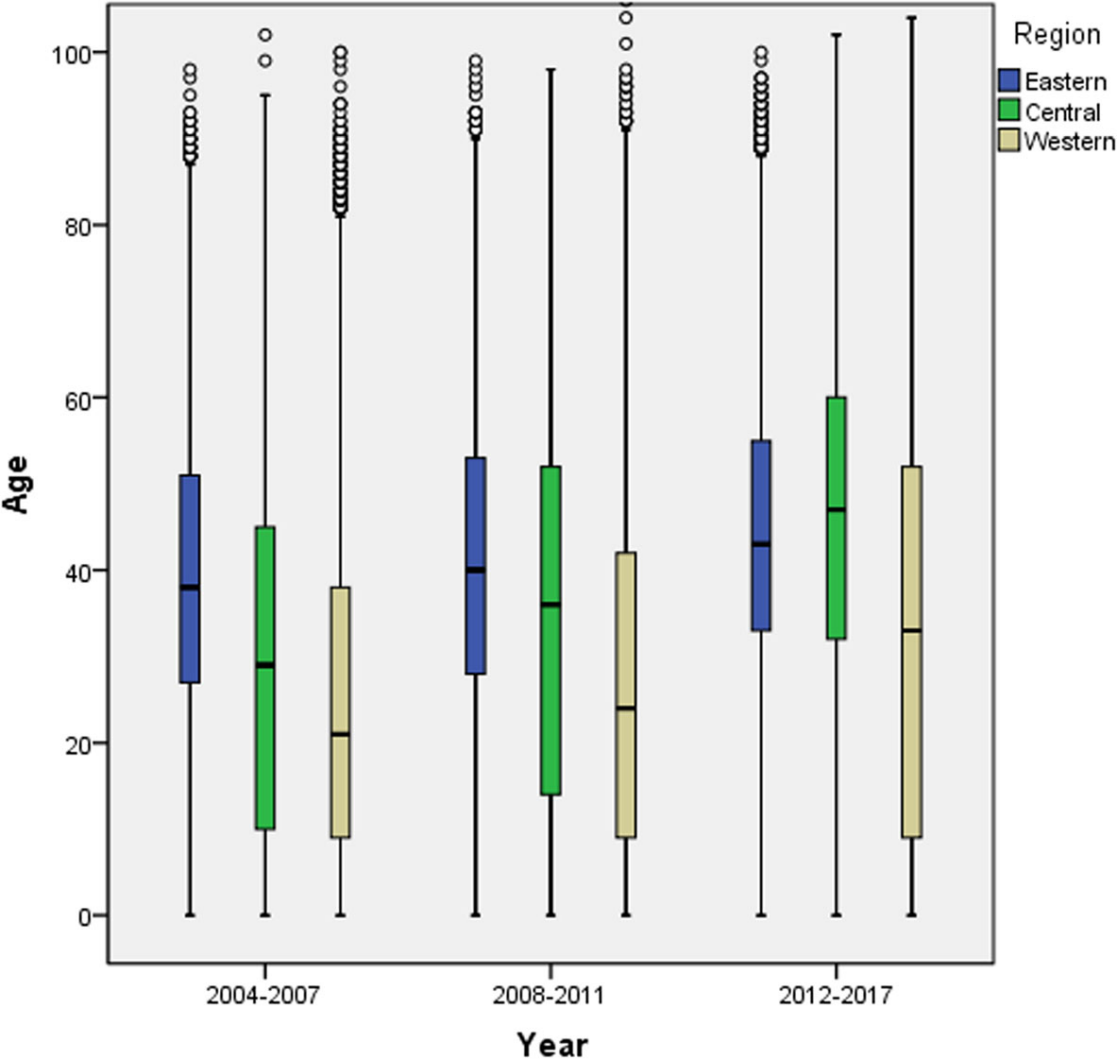
Quartile age of hepatitis A cases in the three regions of China from 2004 to 2017

In 2004–2007, the median ages of HepA cases were 38 (Q_1_–Q_3_: 27–51), 29 (Q_1_–Q_3_: 10–45), and 21 (Q_1_–Q_3_: 9–38) years old in the eastern, central and western regions. In 2008–2011, the median ages increased to 40 (Q_1_–Q_3_: 28–53), 36 (Q_1_–Q_3_: 14–52) and 24 (Q_1_–Q_3_: 9–42). In 2012–2017, the median ages further increased to 43 (Q_1_–Q_3_: 33–55), 47 (Q_1_–Q_3_: 32–60) and 33 (Q_1_–Q_3_: 9–52) years old. Compared with 2004–2007, the median age increased by five, 18, and 12 years, respectively, while the interquartile range enlarged from 29 to 43 in 2012–2017 in the western region while it decreased from 35 to 28 in the central region (Fig. 3).

### Seasonality of HepA from 2004 to 2017

**Fig. 4:**
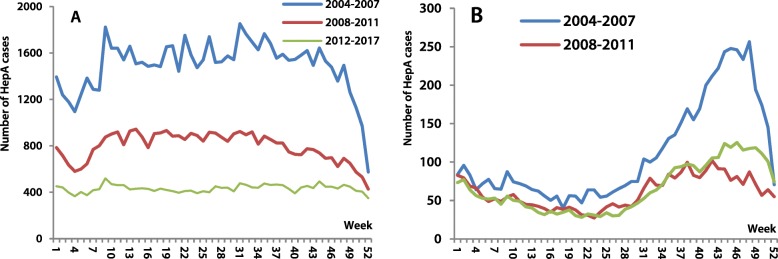
Number of hepatitis A cases by week from 2004 to 2017 in China. Note: A is Nationwide, B is Xinjiang

The national, weekly mean number of HepA cases decreased from 1493 to 795 and 432 in 2004–2007, 2008–2011, 2012–2017, respectively, while the seasonality intensity value of HepA was 17.1, 18.6 and 15.0, indicating low seasonal intensity. The national trend of seasonality was also seen in the three regions, although there was evidence of seasonality in some provinces/autonomous regions, including Xinjiang (intensities of 36.02, 22.04, 31.15 respectively), showing autumn-winter peaks. (Fig. 4).

### Coverage and incidence

**Fig. 5:**
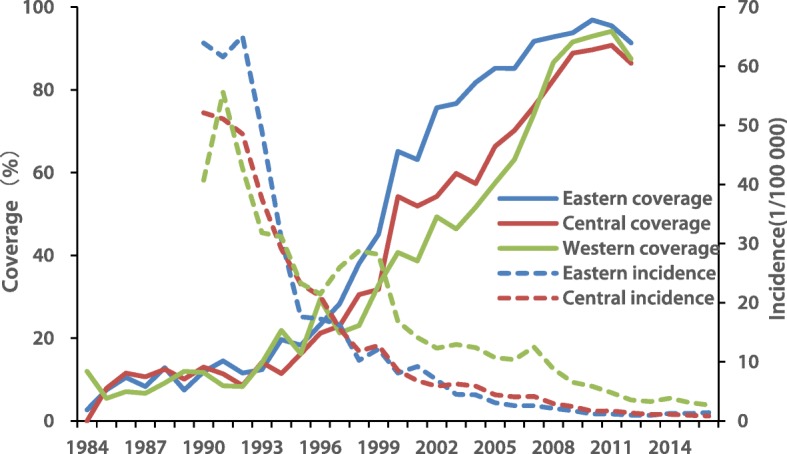
The coverage of hepatitis A vaccine among people born between 1985 and 2012 in China

HepA vaccine coverage was less than 15% among people born before 1994 in all three regions, but increased among those born after 1994 reaching 75.7, 54.3%s and 49.3% in eastern, central, and western regions among people born in 2002. In the early years before Hep A vaccine was incorporated into the EPI system (1984–2007), coverage in eastern region (46.6, 95% *CI*: 44.7–48.5) was higher than in the central (37.2, 95% *CI*: 35.3–39.0) and western regions (32.9, 95% *CI*:31.1–34.6). Since 2008, coverage has been > 80% in all three regions (Fig. 5).

There was an inverse relation between coverage and incidence in eastern (F = 45.40, *P* < 0.01), central (F = 51.40, *P* < 0.01), and western (F = 64.66, *P* < 0.01) regions, with coefficients of determination being 68.37, 70.80, and 75.49%.

## Discussion

Before the availability of HepA vaccine in 1992, the HepA incidence was highest in the eastern region, which had a higher GDP per capita and better sanitation and drinking water [14]. This could be related to lower sero-protection after natural infection of young children, which tends to be subclinical, leading to a larger population of older, susceptible individuals who were more likely to have clinical (and therefore reported) disease. This was determined to be the case in a hepatitis sero-survey conducted in 1992 [15] and was reinforced by the HepA outbreak in 1988 in Shanghai [2]. Since 1993, the year following the licensure of live, attenuated HepAvaccine, the incidence of HepA had been declining in all three regions, but more sharply in the eastern region than the central and western regions. This differential decline in incidence may be partially attributed to higher coverage with HepA vaccine, as the wealthier population of the eastern provinces may have been more willing to pay out of pocket for the vaccine. After HepA vaccine was integrated into the EPI system in 2008, making it free for everyone, regardless of socioeconomic status, the HepA incidence declined to very low levels, and the inequity of disease burden greatly decreased in all three regions [6].

From 2004 to 2017, the HepA incidence had been declining in all age groups in the three regions, especially notable in young children who were covered by EPI. At the same time, a declining incidence in the elder population was observed, suggesting indirect protection. The possible impact of EPI was also observed in other countries, including the United States [16] and Israel [17]. However, the incidence was always high in the younger age groups (0–9 years) in the western region while the higher incidence shifted from younger age groups to older age groups (population ≥ 60) in the central region. Reasons for this phenomenon include that in the western region, timely vaccination with HepA vaccine was low at 18 months when children were mostly susceptible to HAV [18] infection due to HAV circulation in the community [18, 19], failure to vaccinate [20], or vaccine failure [21]. Additional research should be done to clarify the causes, so that effort can be focused on strengthening routine immunization, improving the efficacy of HepA vaccine or promoting better sanitation and safe drinking water.

The median age of HepA cases increased over time – a phenomenon that has been seen in other studies [4, 22]. The likelihood of symptomatic cases increases with age, suggesting that exposure to HAV at older age might be associated with an increase in HepA morbidity and a greater propensity for visible outbreaks [23]. Evidence for this phenomenon can be demonstrated by shifting seroprevalence. Compared with 1992, the seroprevalence of anti-HAV in 2014 declined by 30% among 20–29 years (Chinese Center for Disease Control and Prevention, unpublished data). Despite the declining HepA incidence, the increased median age of HepA cases underlines the importance of continued surveillance for HAV. Surveillance is essential for preparation and response to outbreaks.

The seasonality of HepA decreased, and was possibly eliminated nationwide - something that has been shown in other studies [8, 10]. However, we observed that some provinces still had annual cyclic recurrences, including Xinjiang [24] in the west and Liaoning [25] in the east. In Xinjiang, there were autumn-winter peaks, whereas in Liaoning, a winter-spring peak usually began after the Spring Festival. Unlike the September to October peaks in Europe, from people returning from holidays and family visits in endemic countries [26], the seasonal peaks in some areas of China were more likely to be caused by ingestion of contaminated food or water, or by infrequent hand washing [27, 28]. Environmental studies need to be conducted to investigate the contribution of food or water contamination with HAV in these areas to reduce or eliminate recurrent HepA outbreaks.

HepA vaccine coverage in the three regions was generally low before HepA vaccine was incorporated into EPI, but rapidly increased to very high levels after EPI introduction, similar to other EPI vaccines in China [11, 29]. The incidence of HepA declined much deeper in the eastern region, which attained higher HepA vaccine coverage. The socioeconomic-regional inequality in HepA vaccine coverage was almost eliminated during the study years, but the impact of EPI on the HepA incidence among children still varied [20], suggesting an inequality related to EPI – perhaps surveillance [18, 30] or capacity building [31]. To achieve a uniformly low incidence of HepA disease, the government should pay attention to disadvantaged areas, especially in the less economically develop western provinces/ autonomous regions. In our study, we observed a strong, negative correlation between coverage and incidence, indicating a positive impact of EPI to change the epidemiology of HepA. This finding could further support implementation of massive HepA vaccination in other developing countries that have transitional economies.

Additional research is needed to eliminate HepA as a public health threat in China. First, the exposure source of HepA outbreaks and sporadic cases should be determined [27] (including vaccination records) so that common sources of infection can be prevented. Second, the high incidence in some areas, especially among children, should be studied to determine whether a high incidence can be attributed to failure to vaccinate or vaccine failure. Third, though live attenuated and inactivated HepA vaccine showed similar effectiveness in protecting people [6], the ability of live attenuated HepA vaccine to shed virus and cause infections among susceptible individuals should be studied [32, 33]. Fourth, given that the evolution of HAV is consistent with laboratory and epidemiological evidence, it is critically important to study the seroprevalence of HAV in China to determine susceptible populations with increased likelihoods of HAV outbreaks.

### Limitations

There are several limitations to our study. First, the vaccination status of adults born before 2002 was based on recall; therefore, the age of vaccination usually could not be unambiguously determined. Second, NNDRS is a passive surveillance system, which can be affected by diagnostic and reporting criteria. Third, regional classification was based on the economy of the 1980s, and the GDP per capita of certain provinces in middle or western regions may well be higher than some provinces in the eastern region. However, our study was based on a regional analysis and the overall socio-economic development in eastern region has always been better than central and western region.

A strength of our study is that it is national in scope and used a long-lasting surveillance platform for determining HepA incidence. Diagnostic criteria were stable through the study period. Coverage was determined from a field survey, which provides a good estimate of true coverage.

## Conclusions

The incidence of HepA changed from high levels to very low levels in China, and the socio-economic inequity in coverage of HepA vaccine was almost eliminated after introduction of HepA vaccine into EPI. However, inequity in the incidence of HepA continue to exist in the western region. More attention should be paid to EPI performance in lower socio-economic development regions.

## Additional files

Additional file 1: Multilingual abstracts in the five official working languages of the United Nations. (PDF 355 kb)

Additional file 2: Changes in the epidemiology of hepatitis A in three socio-economic regions of China, 1990–2017. (DOCX 632 kb)

## Data Availability

The datasets used or analysed for this study are available from the corresponding author upon request.
