# Supplementary material for: Changes in the epidemiology of hepatitis A in three socio-economic regions of China, 1990–2017
Source: Infect Dis Poverty. 2019 Oct 3;8:80. doi: 10.1186/s40249-019-0591-z (PMC6775660; doi:10.1186/s40249-019-0591-z)
Supplement: Supplementary file 1 — Multilingual abstracts in the five official working languages of the United Nations. (PDF 355 kb) [file 40249_2019_591_MOESM1_ESM.pdf]

Translation of the abstract into the five official working languages of the United Nations

التغيرات في وبائيات الالتهاب الكبدي أ في ثلاث مناطق اجتماعية واقتصادية في الصين ما بين سنوات 1990 –2017

شياو جين صن ، قوه مين تشانغ ، رونغ جون تشو ، هوى تشنغ ، نينغ مياو ، زون دونغ يين ، فو تشن وانغ

الملخص
المعلومات الأساسية: إن التلقيح ضد التهاب الكبد أ والتحولات الاقتصادية يمكن أن تغير علم وبائيات التهاب الكبد. فقد كان متعارفاً عليه أن الناتج المحلي الإجمالي للفرد في الصين يرتبط بشكل عكسي مع تفشي التهاب الكبد أ ، لكن لازال يتطلب فهم وبائيات هذا الداء بصورة أعمق في مختلف المناطق الاجتماعية والاقتصادية. نقارن تغير وبائيات التهاب الكبد أ في ثلاث مناطق جغرافية اجتماعية واقتصادية في الصين.
الطرق: لقد حصلنا على معلومات تتعلق بجميع حالات التهاب الكبد أ المبلغ عنها من خلال النظام الوطني للإبلاغ عن الأمراض وقمنا بتقييم الاتجاهات وتغيرات معدلات الإصابة المحددة عمرياً حسب الربع العمري وكذا الموسم. قمنا بتصنيف البلد إلى ثلاث مناطق ، وهي السنوات المتتالية إلى خمسة عصور ، مقارنةً بالحدوث والعمر الربعي والكثافة الموسمية وتفشي الداء في المناطق الثلاث. تم إجراء الانحدار الخطي لتحليل اتجاهات حدوث داء التهاب الكبد أ وتحليل الارتباط بين انتشاره ووقوعه.
النتائج: انخفض معدل الحوادث السنوية للداء في المناطق الشرقية والوسطى والغربية من 63.52 / 100000 و 50.57 / 100000 و 46.39 / 100000 في 1990-1992 إلى 1.18 / 100000 و 1.05 / 100000 و 3.14 / 100000 في 2012–2017 ، على التوالي. وشوهدت حالات انخفاض في جميع الفئات العمرية في المناطق الثلاث ؛ حيث كانت النسبة الأعلى (9.3 / 100000) في الفئة العمرية الأصغر (0-4 سنوات) بالمنطقة الغربية ، بينما في المنطقة الوسطى ، تغيرت الفئة العمرية ذات أعلى نسبة إصابة من 0 إلى 9 سنوات إلى البالغين أكثر من 60 سنة. في عام 2017 ، كان متوسط عمر حالات الداء المشخصة هو 43 عاماً (Q1–Q3: 33–55) و 47 عاماً (Q1–Q3: 32–60) و 33 عاماً (Q1–Q3: 9–52) في المقاطعات الشرقية والوسطى والغربية ، على التوالي. لم تعد تصل الذروة الموسمية لما كانت عليه من قبل لا بل يمكن القول أنها على وشك الاندثار على مستوى البلاد ، إلا في بعض المقاطعات. بعد أن شمل البرنامج الموسع للتحصين (EPI) لقاح الالتهاب الكبدي أ في الجدول الروتيني عام 2007 ، زادت تغطية الداء إلى أكثر من 80 ٪ في المناطق الثلاث وارتبطت سلباً مع حالات الالتهاب الكبدي أ التي تم تشخيصها.
الاستنتاج: انخفض معدل حدوث الالتهاب الكبدي أ بشكل ملحوظ بين أعوام 1990 و 2017. قضي على عدم المساواة الاجتماعية والاقتصادية في تغطية لقاح داء الالتهاب الكبدي أ بعد إدخال اللقاح في نظام التحصين الموسع في الصين ، ولكن لا زال هنالك تباين في حدوثه في المنطقة الاجتماعية الأقل تقدماً.

Translated from English version into Arabic by Riyad Zouag, Revised by Amal Imam, through

TRANSLATORS
WITHOUT BORDERS

1990–2017 年中国三个社会经济发展地区的甲型肝炎流性特征变化

摘要
引言：甲型肝炎疫苗接种及经济转型可改变甲型肝炎的流性特征。中国人均 GDP 与甲型肝炎发病率呈负相关，但缺乏甲型肝炎在不同经济发展程度地区内的流行特征深入分析。本文比较了中国三个社会经济发展地区的甲型肝炎流行特征。
方法：通过国家法定传染病报告系统获得所有甲型肝炎病例信息，分析年龄别发病率、病例四分位年龄、季节性。同时分析甲型肝炎发病率与疫苗接种率的关系。

结果：中国东部、中部、西部地区的甲型肝炎年平均发病率分别从 1990–1992 年的 63.52/100 000, 50.57/100 000 和 46.39/100 000，下降至 2012–2017 年的 1.18/100 000, 1.05/100 000 和 3.14/100 000。三个地区的各年龄组发病率均有下降，西部地区的 0–4 岁儿童发病率最高（9.3/100 000），而中部地区发病率最高人群则由 0–9 岁变为≥60 岁人群。2017 年，甲型肝炎病例的中位年龄在东部、中部、西部地区分别为 43 岁 (Q1-Q3:33–55), 47 岁 (Q1-Q3:32–60) 和 33 岁 (Q1-Q3:9–52)。全国范围内的季节性高峰越来越小，但在一些省份仍然存在。甲型肝炎疫苗在 2007 年纳入扩大免疫规划以后，疫苗接种率在三个地区均上升至>80%，且与发病率呈负相关。
结论：1990–2017 年甲型肝炎发病率下降明显，社会经济不均衡导致的甲型肝炎疫苗接种率差异几乎被消除，但社会经济发展较弱的地区仍然存在发病率的差异。

Évolution de l'épidémiologie de l'hépatite A dans trois régions socio-économiques chinoises, 1990-2017

Xiao-Jin Sun, Guo-Min Zhang, Rong-Jun Zhou, Hui Zheng, Ning Miao, Zun-Dong Yin, Fu-Zhen Wang

Résumé

Contexte : La vaccination et les transitions économiques peuvent modifier l'épidémiologie de l'hépatite A. On sait que l'incidence de l'hépatite A est inversement proportionnelle au produit intérieur brut (PIB) de la Chine mais l'épidémiologie de l'hépatite A reste insuffisamment étudiée dans plusieurs régions socio-économiques du pays. Nous comparons l'évolution de l'épidémiologie de l'hépatite A dans trois régions socio-économiques et géographiques de Chine.
Méthodes : Nous avons obtenu des données sur tous les cas d'hépatite A déclarés par le Système national de signalement des maladies à déclaration obligatoire et évalué les variations et l'évolutions des taux d'incidence en fonction de l'âge, de la tranche d'âge (quartile) et de la saison. Nous avons divisé le pays en trois régions et les années successives en cinq périodes, pour comparer l'incidence de l'hépatite A, les quartiles d'âge, l'intensité saisonnière et la couverture vaccinale pour les trois régions. Une régression linéaire a été réalisée pour analyser les évolutions de l'incidence de l'hépatite A et pour faire le lien entre couverture vaccinale et incidence.
Résultats : L'incidence moyenne annuelle des hépatites A dans les régions de l'est, du centre et de l'ouest a diminué, passant respectivement de 63,52/100 000, 50,57/100 000 et 46,39/100 000 en 1990-1992 à 1,18/100 000, 1,05/100 000 et 3,14/100 000 en 2012-2017. Une baisse de l'incidence a été observée dans toutes les tranches d'âge et dans les trois régions ; l'incidence la plus élevée (9,3/100 000) concerne la tranche d'âge la plus jeune (0–4 ans) de la région ouest, tandis que dans la région centrale, la tranche d'âge dont l'incidence était la plus élevée est passée de 0–9 ans à ≥ 60 ans. En 2017, l'âge médian des cas d'infection par l'hépatite A était respectivement de 43 ans (Q_1–Q_3 : 33–55), 47 ans (Q_1 Q_3 : 32–60) et 33 ans (Q_1–Q_3 :9–52), dans les régions de l'est, du centre et de l'ouest. Les pics saisonniers ont diminué et ont quasiment disparu à l'échelle nationale, mais la saisonnalité persiste dans certaines provinces. Après que le vaccin contre l'hépatite A a été inclus dans le calendrier vaccinal du Programme élargi de vaccination (PEV) en 2007, la couverture vaccinale est passée à 80 % dans les trois régions, ce qui est associé à une baisse de l'incidence de l'hépatite A.

Conclusion : L’incidence de l’hépatite A a nettement diminué entre 1990 et 2017. Les inégalités socio-économiques dans la couverture vaccinale ont pratiquement disparu avec l’introduction du vaccin contre l’hépatite A dans le système du PEV en Chine, mais il reste des inégalités d’incidence dans les régions de faible développement socio-économique

Translated from English version into French by Sarah Merlin, Revised by Suzanne Assenat, through

TRANSLATORS WITHOUT BORDERS

Изменения в эпидемиологии гепатита А в трех социально-экономических регионах Китая, 1990–2017 гг.

Сяо-Цзинь Сунь, Го-Минь Чжань, Жун-Цзюнь Чжоу, Хуй Чжэн, Нин Мяо, Цзунь-Дун Инь, Фу-Чжэнь Ван

Аннотация

Справочная информация: Вакцинация против вирусного гепатита А (ВГА) и экономические изменения могут повлиять на эпидемиологию ВГА. Известно, что валовой внутренний продукт (ВВП) Китая на душу населения обратно пропорционально связан с распространенностью ВГА, однако глубокое понимание эпидемиологии ВГА в различных социально-экономических регионах отсутствует. Мы сравниваем изменяющуюся эпидемиологию ВГА в трех социально-экономико-географических регионах Китая.

Методы: Мы получили данные по всем случаям ВГА, которые были учтены в Национальной системе отчетности по болезням, подлежащим регистрации, и оценили тенденции и изменения в показателях заболеваемости в зависимости от возрастных квартилей и времени года. Мы классифицировали страну на три региона, последовательные годы на пять периодов, сравнили заболеваемость, возрастные квартили, сезонную интенсивность и охват ВГА в трех регионах. Для анализа тенденций заболеваемости ВГА и связи между охватом и заболеваемостью была использована линейная регрессия.

Результаты: Среднегодовая заболеваемость ВГА в восточном, центральном и западном регионах снизилась с 63,52/100 000, 50,57/100 000 и 46,39/100 000 в 1990-1992 годах до 1,18/100 000, 1,05/100 000 и 3,14/100 000 в 2012-2017 годах, соответственно. Снижение заболеваемости было отмечено во всех возрастных группах в трех регионах; самая высокая заболеваемость (9,3/100 000) - в самой молодой возрастной группе (0–4 года) в западном регионе, в то время как в центральном регионе возрастная группа с самой высокой заболеваемостью изменилась с 0–9 лет на возраст старше 60 лет. В 2017 году средний возраст случаев ВГА составил 43 года (Q_1–Q_3: 33–55), 47 лет (Q_1–Q_3: 32–60) и 33 года (Q_1–Q_3: 9–52) в восточной, центральной и западной провинциях соответственно. Сезонные вспышки стали меньше или почти исчезли по всей стране, но в некоторых провинциях сезонность сохранилась. После того, как Расширенная программа иммунизации (РПИ) включила вакцину ВГА в рутинный график в 2007 году, охват ВГА увеличился до > 80 % в трех регионах

и привел к снижению заболеваемостью ВГА.
Заключение: Заболеваемость ВГА заметно снизилась в период с 1990 по 2017 год. Социально-экономическое неравенство в охвате вакцинации против ВГА было практически устранено после внедрения вакцины против ВГА в китайскую систему РПИ, однако неравенство в отношении заболеваемости по-прежнему существует в регионе с более низким социально-экономическим развитием.

Translated from English version into Russian by Anna Kukharchuk, Revised by Michael Orlov, through

TRANSLATORS
WITHOUT BORDERS

Cambios en la epidemiología de la hepatitis A en tres regiones socio-económicas de China, 1990–2017

Xiao-Jin Sun, Guo-Min Zhang, Rong-Jun Zhou, Hui Zheng, Ning Miao, Zun-Dong Yin, Fu-Zhen Wang

Resumen
Antecedentes: la vacunación contra la Hepatitis A (HepA) y las transiciones económicas pueden cambiar la epidemiología de la HepA. Se conoció que el Producto Bruto Interno (PBI) per cápita de China estuvo inversamente asociado a la incidencia de la HepA, pero hace falta un entendimiento más profundo de la epidemiología de la HepA en diferentes regiones socioeconómicas. Comparamos la cambiante epidemiología de la HepA en tres regiones socioeconómicas geográficas de China.
Métodos: obtuvimos información de todos los casos de HepA reportados por el Sistema Nacional de Reporte de Enfermedades Notificables y evaluamos las tendencias y cambios en las tasas de incidencia en edad específica por cuartil de edad y temporada. Clasificamos el país en tres regiones, los años secuenciales en eras de cinco años, comparamos la incidencia, el cuartil de edad, la intensidad estacional y la cobertura de la HepA de las tres regiones. Se llevó a cabo una regresión lineal para analizar las tendencias en la incidencia de la HepA y analizar la asociación entre cobertura e incidencia.
Resultados: el promedio anual de incidencias de la HepA en las regiones oriental, central y occidental disminuyó de 63,52/100.000; 50,57/100.000; y 46,39/100.000 en 1990–1992 a 1,18/100.000; 1.05/100.000; y 3,14/100.000 en 2012–2017, respectivamente. Las disminuciones en la incidencia se vieron en todos los grupos de edad en las tres regiones; la incidencia fue más alta (9,3/100.000) en el grupo de menor edad (0–4 años) de la región occidental, mientras que en la región central el grupo de edad con la incidencia más alta cambio de 0–9 años a adultos ≥ 60 años de edad. En 2017, la edad promedio de casos de HepA era 43 años (Q_1–Q_3: 33–55), 47 años (Q_1–Q_3: 32–60) y 33 años (Q_1–Q_3: 9–52) en las regiones oriental, central y occidental, respectivamente. Los puntos máximos estacionales se volvieron más pequeños o fueron casi eliminados a nivel

nacional pero la estacionalidad persistió en algunas provincias. Después de que el Programa Extendido de Inmunización (PEI) incluyó la vacuna contra la HepA en su programa de rutina en 2007, la cobertura contra la HepA aumentó a > 80 % en las tres regiones y fue negativamente asociada con la incidencia de la HepA.

Conclusión: la incidencia de HepA disminuyó marcadamente entre 1990 y 2017. La desigualdad socioeconómica en la cobertura de la vacuna contra la HepA fue casi eliminada después de que la vacuna contra la HepA fuera incluida en el sistema PEI de China, pero la desigualdad en la incidencia continuó existiendo en regiones con bajo desarrollo socioeconómico.

Translated from English version into Spanish by Ilduara Escobedo, Revised by Gabriel Cano, through

TRANSLATORS
WITHOUT BORDERS
