# Supplementary material for: Changes in the epidemiology of hepatitis A in three socio-economic regions of China, 1990–2017
Source: Infect Dis Poverty. 2019 Oct 3;8:80. doi: 10.1186/s40249-019-0591-z (PMC6775660; doi:10.1186/s40249-019-0591-z)
Supplement: Supplementary file 2 — Changes in the epidemiology of hepatitis A in three socio-economic regions of China, 1990–2017. (DOCX 632 kb) [file 40249_2019_591_MOESM2_ESM.docx]

**Changes in the epidemiology of hepatitis A in three socio-economic regions of China, 1990**‒**2017**

**Technical appendix**

**Three regions in China**


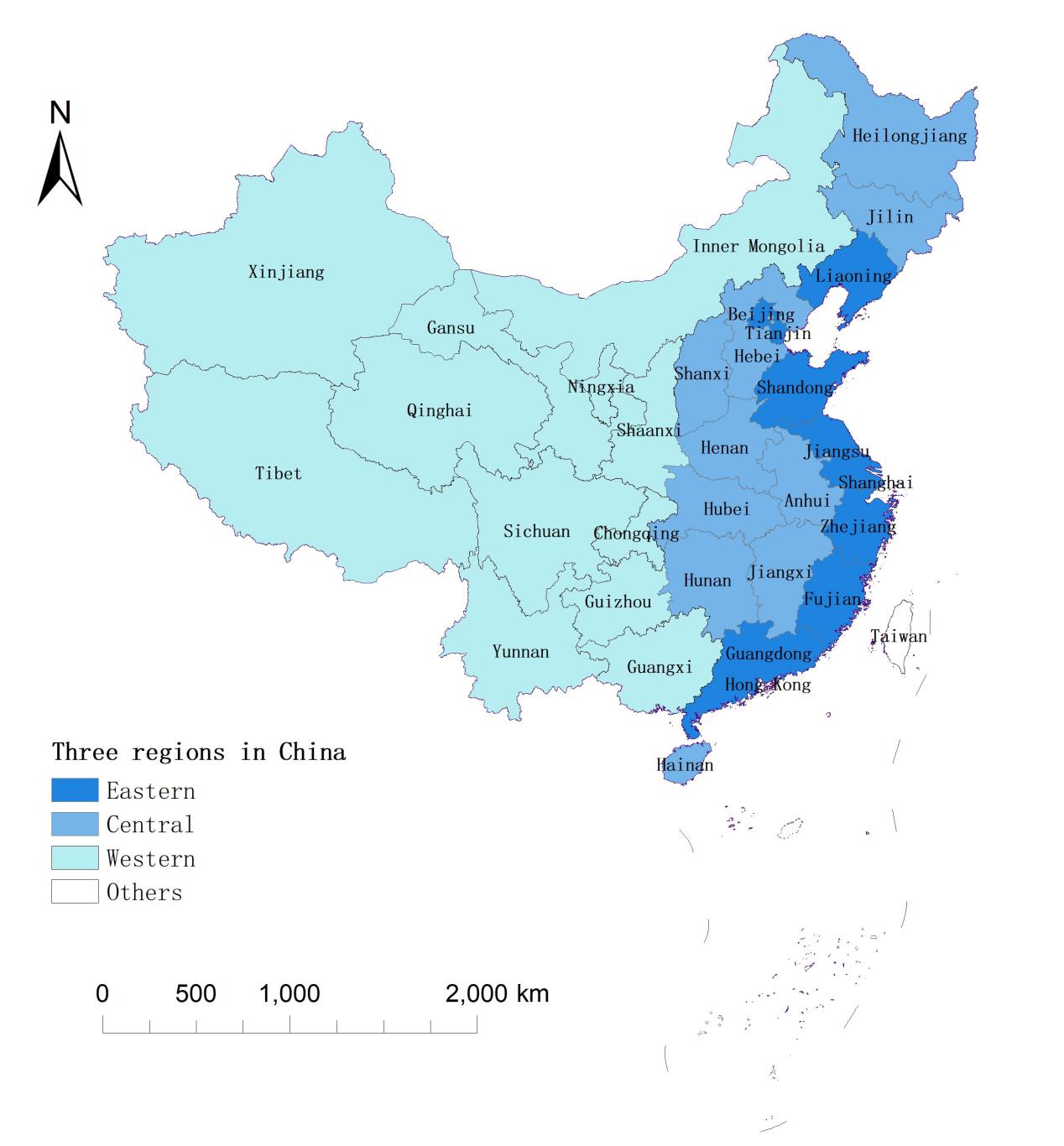


Technical appendix fig1. Three regions were divided, with Eastern region including 9 higher developed provinces of Beijing, Tianjin, Liaoning, Shandong, Jiangsu, Shanghai, Zhejiang, Fujian and Guangdong, Central region including 10 provinces of Heilongjiang, Jilin, Hebei, Henan, Shanxi, Hubei, Hunan, Jiangxi, Anhui and Hainan, Western region including 12 lower developed provinces of Inner Mongolia, Guangxi, Chongqing, Sichuan, Guizhou, Yunnan, Tibet, Shaanxi, Gansu, Qinghai, Ningxia and Xinjiang. Data of HepA incidence was not available in Hongkong SAR, Macau SAR and Taiwan province.
